# Janus gold nanoparticles obtained *via* spontaneous binary polymer shell segregation†
†Electronic supplementary information (ESI) available: Experimental procedures, results of SAXS, UV-vis and DLS for NPs of different core sizes, polymer coatings and in different solvents; details of emulsification using Janus Au NPs; TEM images after silica coating of Janus Au NPs; 3D images of different stained Au NPs. See DOI: 10.1039/c5cc10454h



**DOI:** 10.1039/c5cc10454h

**Published:** 2016-02-09

**Authors:** Ana M. Percebom, Juan J. Giner-Casares, Nathalie Claes, Sara Bals, Watson Loh, Luis M. Liz-Marzán

**Affiliations:** a CIC BiomaGUNE , Paseo de Miramón 182 , 20009 Donostia-San Sebastián , Spain . Email: llizmarzan@cicbiomagune.es; b Institute of Chemistry , University of Campinas (UNICAMP) , CP 6154 Campinas , SP , Brazil; c Department of Chemistry , Pontifícal Catholic University of Rio de Janeiro , 22451-900 , Rio de Janeiro , RJ , Brazil . Email: apercebom@puc-rio.br; d Biomedical Research Networking Center in Bioengineering , Biomaterials, and Nanomedicine, CIBER-BBN , Spain; e EMAT-University of Antwerp , Groenenborgerlaan 171 , B-2020 Antwerp , Belgium; f Ikerbasque , Basque Foundation for Science , 48013 Bilbao , Spain

## Abstract

Janus gold nanoparticles are of high interest because they allow directed self-assembly and display plasmonic properties. We succeeded in coating gold nanoparticles with two different polymers that form a Janus shell. The spontaneous segregation of two immiscible polymers at the surface of the nanoparticles was verified by NOESY NMR and most importantly by electron microscopy analysis in two and three dimensions. The Janus structure is additionally shown to affect the aggregation behavior of the nanoparticles.

## Abstract 2

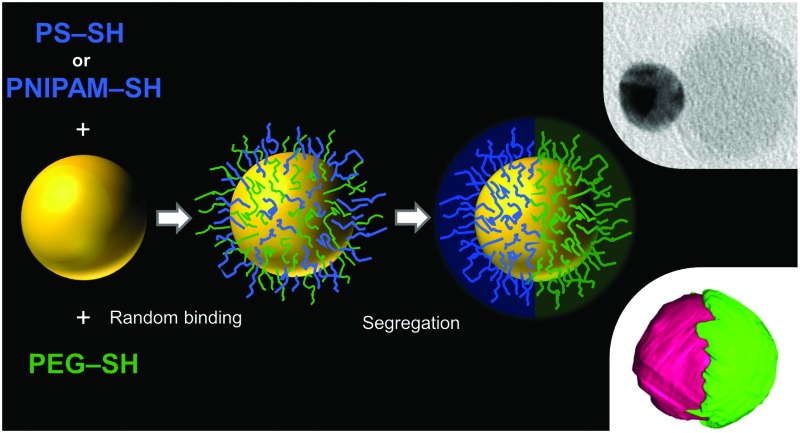
The spontaneous formation of a Janus polymer shell is clearly demonstrated by electron tomography and NOESY-NMR.

Nanoparticles (NPs) displaying two domains with different chemical natures are termed “Janus NPs”, with reference to the name of the Roman god Janus, who had two faces. Janus NPs can be considered as amphiphilic nanoparticles, which can self-assemble or even form hierarchical structures over different length scales.^1–3^ The collective properties of assembled Janus NPs have been proposed as potential solutions in different areas. Indeed, Janus NPs often carry multiple properties, which is of interest toward a variety of technologies such as catalysis,^4^ emulsion stabilization,^5^ sensing,^6^ biomarking^7^ and drug delivery.^8^ When Au NPs are used as cores, Janus NPs can also display localized surface plasmon resonances (LSPR), thus providing the opportunity to tune collective optical effects arising from plasmon coupling in NP assemblies.^9^ Therefore, Janus Au NPs can offer tuneable assembly, remarkable optical features and include other functionalities within a single NP.

Currently, demonstration of properties and applications of Janus NPs is still hindered because of the lack of simple synthetic and characterization methods that can unambiguously verify their formation and structure.^10^ Intensive research efforts have been currently devoted in this direction. Liquid/liquid interfaces have been used to accommodate metallic NPs and perform different chemical reactions in either phase.^11^ Due to the mobility of different thiol-terminated ligands chemisorbed on gold surfaces, immiscible ligands can self-assemble into separate domains.^12^ Stellacci *et al.* synthesized spherical Au NPs of different sizes coated with different combinations of two unalike ligands.^13–15^ Au NPs with diameters between 3 and 8 nm were coated by two immiscible ligands, forming stripe-like domains,^13^ whereas Janus shells were reported for NPs smaller than 1.5 nm in diameter. The characterization of ligand segregation has been mainly performed using scanning tunnelling microscopy and nuclear Overhauser effect spectroscopy (NOESY) NMR,^15^ as well as simulations predicting phase separation of the ligands into a Janus pattern for very small NPs.^16^ Coating with unalike ligands was also applied to Au NPs up to 7 nm in diameter.^17,18^ Interestingly, mass spectrometry was used to estimate ligand segregation in 5 nm Au NPs.^19^

A characterization method for the unambiguous determination of an organic Janus shell structure is however still missing. Additionally, a major limitation of the current synthetic methods to obtain Janus NPs is the limited size of the metal cores. We propose that the use of polymer ligands with longer chain lengths should be a suitable strategy to promote phase segregation into a Janus configuration at the surfaces of larger Au NPs. To date, only methods involving polymer synthesis (grafting-from) and templating (grafting-to) have been reported.^20,21^ We present a general synthetic method to prepare larger Au NPs with Janus character, based on the use of two immiscible thiolated polymers, which segregate into a bicompartment shell. We use a single step, one phase method not requiring the use of templates, chemical reactions (other than thiol–gold bonding), or further polymeric modifications. The obtained Janus NPs present interesting self-assembly properties. This synthetic method can be applied to Au cores of different sizes and coating polymers of varying chain lengths. Most interestingly, the true Janus character of the NPs was unambiguously determined by electron tomography with specific staining of the polymer hemispheres.

The mixing entropy in solution is lower for polymers than for the corresponding monomers, so that phase separation may actually occur for two incompatible polymers, even in a common solvent, at high concentrations.^22^ Janus NPs were prepared by simply adding citrate-capped Au NPs to a dilute solution of two incompatible thiol-terminated polymers in a common solvent. The solution concentration was low enough to dissolve the polymers, but high enough to be in excess with respect to the available gold nanoparticle surface. Thiol groups readily bind to the NP surface, forming a polymer shell. The high local concentration of polymers at the nanoparticle surface leads to segregation in the shell.

Hydrophobic polystyrene (PS) and hydrophilic polyethylene glycol (PEG) in tetrahydrofuran (THF) – a good solvent for both polymers – were used to prepare the first example of Janus NPs by this method. As a control experiment, mixing of PEG-coated and PS-coated Au NPs in water was found to lead to irreversible aggregation, thus showing the requirement of the Janus character to achieve a stable dispersion. Interestingly, the assembly of Janus NPs can be controlled in dispersions using a liquid that is a good solvent for only one of the ligands. Analysis of the assembly of Au NPs with different polymeric coatings in water (good solvent for PEG, bad solvent for PS) is summarized in Fig. S1 (ESI†). 23 nm Au NPs stabilized by citrate feature a plasmon band with maximum absorbance at 520 nm, whereas a slight redshift is observed (525 nm) upon coating with PEG (1 kDa and 5 kDa for PEG1 and PEG5, respectively) and further to 527 nm when coated by PEG5 + PS. Janus Au NPs coated by PEG1 + PS display a significant red shift and broadening of the LSPR band, indicating plasmon coupling due to aggregation. These observations were confirmed by DLS determination of hydrodynamic radii. Assuming spherical clusters, we estimate 120 NPs per cluster for PEG1 + PS, but 8 NPs per cluster only for PEG5 + PS (Fig. S1, ESI†), showing the higher stability provided by the longer chains of PEG5. Janus Au NPs could be transferred to other solvents and the same method could be applied using different polymer ratios. We prepared Janus Au NPs with 13, 23 and 33 nm Au cores dispersed in water and THF (Fig. S1 and S2, ESI†), as well as varying the PEG content from 50% to 100% (Fig. S3, ESI†). Thus, various experimental parameters can be tuned to affect the NP self-assembly, leading to the formation of clusters with different sizes, in analogy to surfactant self-assembly, where either the hydrophobic chain length or the polar head size can be varied. The extent of the assembly was additionally confirmed by measuring the structure factor in SAXS (Fig. S4 and Table S1, ESI†) and Janus NPs can form chloroform/water emulsions, see Fig. S5 and S6 (ESI†).

A more interesting application of this synthesis method was carried out by coating NPs with two polymers that are both hydrophilic at room temperature, PEG and poly(*N*-ispropylacrylamide, PNIPAM), but the latter shows a hydrophilic to hydrophobic transition at a lower critical solution temperature (LCST) of 32 °C.^23^ Janus NPs containing PEG + PNIPAM should therefore self-assemble upon heating but polymer segregation on the NP surface can occur even at RT, in analogy to the PEG-*b*-PNIPAM free polymer in bulk solution at temperature values below the LCST.^24^ Both Au NPs coated by only PNIPAM and with the mixture PEG + PNIPAM could be dispersed in water at 25 °C, as expected from the hydrophilic nature of both polymers (Fig. S7, ESI†). A temperature increase leads to an LSPR redshift in both cases, due to the self-assembly induced by the PNIPAM transition into a hydrophobic phase above 32 °C. The LSPR shift was slightly larger for PNIPAM-coated AuNPs, but in both cases the LSPR position could be recovered when the temperature was lowered again over several cycles (Fig. S8, ESI†).

Polymer segregation on the surface of Janus AuNPs was demonstrated by NOESY 2D NMR spectroscopy. Cross-peaks are observed in the case of effective dipole–dipole interactions between nearby nuclear spins.^25^ The NOESY spectra for PEG + PS and PEG + PNIPAM Janus AuNPs are shown in Fig. 1, indicating a significant segregation of the polymers on the NP surface. It should be noted that these data do not confirm whether segregation occurs into a Janus shell or into a different patchy conformation.

**Fig. 1:**
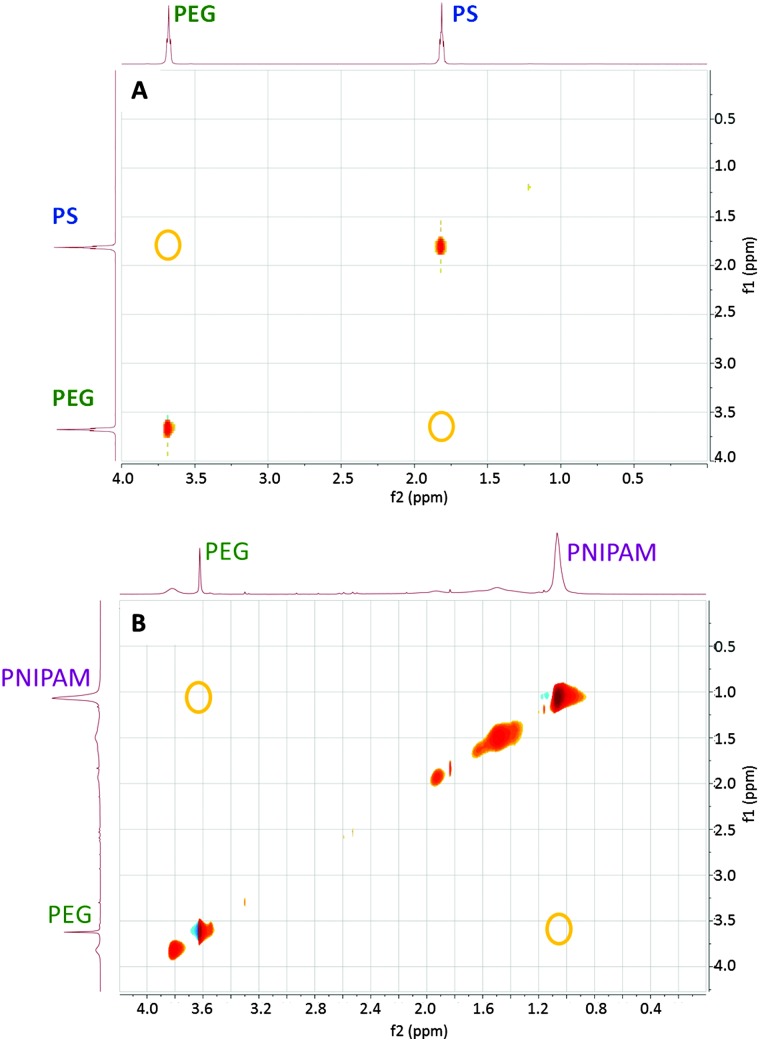
2D ^1^H–^1^H NOESY NMR spectra of gold nanoparticles coated by: (top) PEG 1 kDa + PS 2 kDa and dispersed in CHCl_3_; (bottom) PEG 1 kDa + PNIPAM 1.2 kDa and dispersed in water. Yellow circles indicate positions where cross-peaks corresponding to the chemical shifts of the most intense peak for each polymer would be expected, in case of cross-correlation.

Characterization of the Janus character of the polymer shell by electron microscopy is challenging due to the low electron absorption by organic materials and the low contrast between PEG and both PS and PNIPAM. We used two alternative approaches to enhance the contrast between the two polymers for TEM. PNIPAM-coated AuNPs showed damage of the polymer layer upon electron beam irradiation for a few seconds. However, for PEG-coated AuNPs the shell was stable enough to survive electron tomography analysis, even though it requires acquisition with multiple exposures (Fig. S9 and S10, ESI†). PEG + PNIPAM Janus AuNPs were thus stained with copper sulphate in an attempt to distinguish PNIPAM from PEG. Although 3D reconstructions based on a tilt series displayed rather low contrast between the shell and the background (Fig. S11, ESI†), manual segmentation based on orthoslices could be successfully carried out. This treatment provided 3D images that clearly confirm the formation of stained half-shells for Au NPs coated by PEG + PNIPAM (Fig. 2 and Fig. S12–S15, ESI†). Acquisition and reconstruction of a tilt series yielded results as shown in Fig. S15 (ESI†). The 3D reconstructed object shows low contrast between the shell and the background, so manual segmentation was performed based on the corresponding orthoslices. Segmentation in the 3 different directions (*xy*, *yz*, *zx*) confirms the formation of a stained half-shell as indicated in green in Fig. 2 (the outer gray shell is the edge of the volume selected for 3D reconstruction). All samples were prepared at room temperature, demonstrating the Janus character of Au NPs coated by PEG + PNIPAM even before heating. This is to our knowledge the first unequivocal experimental proof of the actual Janus character of coating shells in Au NPs, using BF-TEM tomography, which provides a clear visualization of the 3D distribution of the Au core and the PEG half shell.

**Fig. 2:**
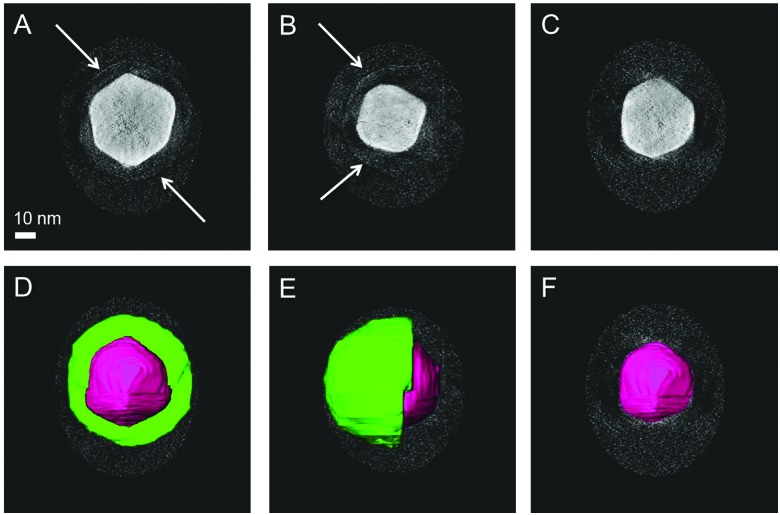
BF-TEM tomography for nanoparticles coated by PEG 1 kDa + PNIPAM 1.2 kDa and stained with CuSO_4_. The tilt series was reconstructed with the SIRT algorithm (Astra Toolbox, see ESI†). Slices through the reconstruction show the absence of a shell (C and F), the presence of a complete shell (A and D) and a half shell (B and E) around the particle (indicated by white arrows) dependent on the position of the slice. In figures D, E and F the segmentation is shown on top of the orthoslices. The scale bar applies to all images.

In the case of PEG + PS AuNPs, selective staining and visualization of one polymer could not be completed. An attempt to use calcium phosphate to stain PEG in PEG + PS Janus Au NPs was carried out. From standard TEM images it appears that only one hemisphere of the Au NPs was coated by a thin layer (of PEG) stained with calcium phosphate, as indicated in Fig. S16 (ESI†). BF-TEM tomography was also used in this case (Fig. S17, ESI†), but selective staining of the PS shell could not be confirmed. Therefore, an alternative approach for indirect visualization of the ligand distribution was carried out, based on the growth of silica over PEG-coated Au NPs. Due to the high affinity of polyethylene glycol by silica, PEG acts as a primer to promote silica condensation onto the Au NP surface, forming a silica shell.^26^ Since PS is hydrophobic and does not allow silica nucleation, shell growth was expected to occur only in the parts of the NPs that are coated by PEG. Fig. 3 and Fig. S18 (ESI†) indeed show the formation of silica half shells over the Au NPs, confirming that PEG and PS segregate forming a bicompartment shell.

**Fig. 3:**
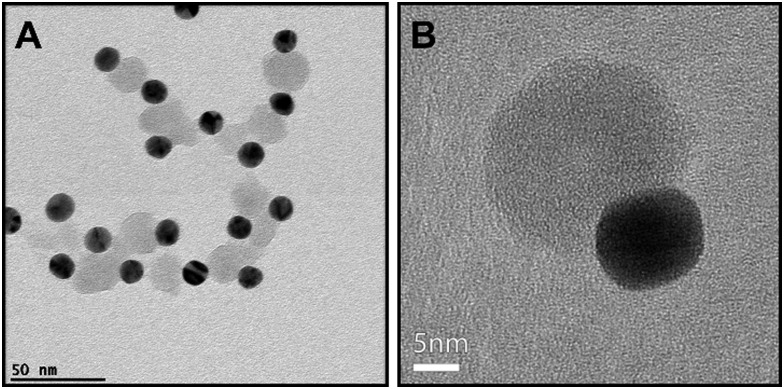
TEM images of 13 nm Au NPs previously coated by PEG 5 kDa + PS 2 kDa after silica growth. A silica half-shell (in gray) has grown over the PEG-coated hemisphere of gold nanoparticles (dark spheres).

We finally tested whether our Janus NPs displayed enhanced cellular membrane permeability compared to hydrophilic NPs, as previously reported for a different system.^27^ We compared the cellular uptake of Janus (PEG + PS) and hydrophilic (PEG) Au NPs. Fig. 4 shows dark-field optical microscopy images of HeLa cells that indicate significantly enhanced uptake of Janus NPs. We propose that the possibility of imposing Janus character on metallic NPs and thereby enhancing the uptake by living cells can have a large impact on the various applications of NPs in biomedicine.

**Fig. 4:**
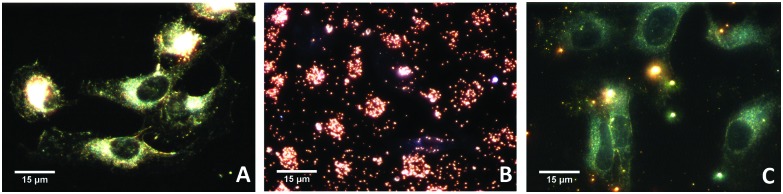
Dark-field images of He-La cells: before incubation (A, control); incubated with Janus gold nanoparticles (B); and incubated with PEG-coated gold nanoparticles (C).

In summary, preparation of true Janus Au NPs has been achieved through a simple one-pot method with common thiol-terminated polymers. The method is based on the segregation between two chemically different polymers, in analogy to small thiolated molecules. The formation of a Janus shell provides amphiphilic character to the NPs, which can therefore self-assemble in a tunable fashion by varying the size, polymer ratio and temperature, among other experimental parameters. Either staining or silica shell growth over PEG-coated areas resulted in the observation of semi-shells in Au NPs by TEM and electron tomography, which may become the technique of choice for other systems. The versatility of the process, the possibilities of controlling self-assembly and of using different polymers render this method promising toward the preparation of a wide range of Janus NPs for different applications.

Funding is acknowledged from the European Research Council (ERC Advanced Grant #267867 Plasmaquo, and ERC Starting Grant #335078 Colouratom). A. M. P. thanks the Brazilian FAPESP for financial support (FAPESP 2012/21930-3 and 2014/01807-8) and J. J. G. C. acknowledges the Spanish MINECO for a Juan de la Cierva fellowship (#JCI-2012-12517). We thank Ada Herrero Ruiz and Daniel Padró for help with NMR measurements, Malou Henriksen-Lacey for cell experiments and the Brazilian Synchrotron Laboratory (LNLS) for allocation of SAXS beamtime.
