# Supplementary material for: Janus gold nanoparticles obtained via spontaneous binary polymer shell segregation
Source: Chem Commun (Camb). 2016 Feb 9;52(23):4278–81. doi: 10.1039/c5cc10454h (PMC5317220; doi:10.1039/c5cc10454h)
Supplement: Supplementary file 1 [file CC-052-C5CC10454H-s001.pdf]

Electronic Supplementary Material (ESI) for Chemical Communications.
This journal is © The Royal Society of Chemistry 2016

Janus Gold Nanoparticles Obtained via Spontaneous Polymer Shell Segregation

Ana M. Percebom,[‡,†,¤,*] Juan J. Giner-Casares,[‡] Nathalie Claes,[«] Sara Bals,[«] Watson Loh,[†] Luis M. Liz-Marzán[‡,§,#,*]

[‡] CIC biomaGUNE, Paseo de Miramon 182, 20009 Donostia-San Sebastián, Spain

[†] Institute of Chemistry, University of Campinas (UNICAMP), CP 6154 Campinas, SP Brazil

[¤] Department of Chemistry, Pontifical Catholic Universitiy of Rio de Janeiro, 22451-900, Rio de Janeiro, Brazil

[«] EMAT-University of Antwerp, Groenenborgerlaan 171, B-2020 Antwerp, Belgium

[§] Ikerbasque, Basque Foundation for Science, 48013 Bilbao, Spain

[#] Biomedical Research Networking Center in Bioengineering, Biomaterials, and Nanomedicine, CIBER-BBN, Spain

1. Materials and Methods
2. Assembly of Janus Au Nanoparticles with different core sizes
3. Chloroform/Water Emulsions stabilized by Janus Au NPs
4. 3D investigation of Janus Au NPs coated by PEG+PS
5. TEM images of Janus AuNPs after silica growth over PEG half shell
6. Uptake of Janus Au NPs by HeLa Cells

1. Materials and Methods

Chemicals

Tetrachloroauric acid ($HAuCl_4 \cdot 3H_2O$), trisodium citrate dehydrate, anhydrous tetrahydrofuran (THF), HPLC-grade chloroform ($CHCl_3$), O-[2-(3-mercaptopropionylamino)ethyl]-O'-methylpoly(ethylene glycol) (PEG-SH 5 kDa), tetraethylorthosilicate (TEOS), and ammonium hydroxide solution (NH_4OH 29%) were purchased from Sigma-Aldrich. Thiol terminated polystyrene (PS-SH 2 kDa), thiol terminated poly(N-isopropylacrylamide) (PNIPAM 1.2 kDa) and thiol terminated poly(ethylene glycol) methyl ether (PEG-SH 1 kDa) were supplied by Polymer Source. Pure-grade ethanol was purchased from Panreac. All chemicals were used as received. Pure-grade ethanol and Milli-Q grade water (resistivity 18.2 MΩ·cm) were used in all preparations. Copper (II) sufate ($CuSO_4.5H_2O$) purchased from BelgoLabo and calcium phosphate powder purchased from Sigma-Aldrich were used as staining reactants.

Gold nanoparticles synthesis

Throughout this study, we mainly used spherical nanoparticles with diameters of up to 33 nm. The method described by Bastús *et. al.* [1] was used for obtaining nanoparticles with sizes of around 20 nm or bigger, by interrupting the synthesis at different steps. For obtaining nanoparticles of slightly smaller sizes, we used the Turkevich method.[2] Both methods results in citrate-stabilized spherical nanoparticles.

Polymer coating, purification and transfer

Solutions of polystyrene and poly(ethylene glycol) in different proportions were prepared in THF in a concentration estimated to have a final proportion of 65 polymer chains per nm^2 of gold surface. The proportion of PEG/PS chains used to prepare the Janus nanoparticles mentioned throughout the text is 50/50, except when clearly stated, but Supporting Information exhibit results for other different proportions from 0/100 to 100/0. Only when the polymer content was 100% of PEG, water was used as solvent during the coating instead of THF. Typically, 0.5 mL of gold nanoparticles with a gold concentration of $[Au^0]$ = 5 mM was added dropwisely to 10 mL of the polymeric solution under vigorous stirring. After 15 min, stirring was interrupted and the mixture was left undisturbed for at least 6 hours previous purification. To remove the excess of polymer, samples were centrifuged at least twice and the supernatant was removed and replaced by pure solvent after each step. The same procedure was used for transferring the nanoparticles to different solvents. The method described was slightly adapted to a second pair of polymers: PEG 1 kDa and PNIPAM 1.2 kDa. For this case, ice-cold water replaced THF as solvent and the samples were centrifuged at 5 ^oC for purification.

Silica growth

To promote silica growth, 13 nm gold nanoparticles coated by PEG 5 kDa and PS 2 kDa were dispersed in an ethanolic mixture with the following final concentrations: [Au]=0.5 mM, $[H_2O]$=10.55 M, $[NH_3]$=0.2 M, and [TEOS]= 0.8 mM, as described by Fernández-López.[3] TEOS was the last reactant added to the mixture under vigorous stirring, followed by gentle stirring maintained overnight. At least two cycles of centrifugation were necessary to remove free silica excess and transfer the nanoparticles to chloroform.

Electron Microscopy

For transmission electron microscopy (TEM), samples were prepared in carbon-coated 400 square mesh copper grids and the images were obtained using a JEO2010F FE-TEM instrument operated at 200 kV and a FEI Tecnai operated at 200 kV.

To generate sufficient contrast in TEM, PEG+PES coated NPs were stained with 3.7 mM calcium phosphate solution and PEG+PNIPAM coated NPs were stained with 3 mM CuSO4.5H2O solution. The staining solution was added to the dispersion of Au NPs coated by the referred polymers. The obtained mixture was stirred and afterwards kept for 2 hours. The mixture was diluted with deionized water and deposited on a TEM grid.

Since conventional TEM images correspond to two-dimensional (2D projections of 3D objects, electron tomography was performed. It must be noted that the conventional technique for tomography in materials science, high angle annular dark field scanning TEM (HAADF-STEM), could not be applied due to the large difference in atomic number Z for Au and the elements in the shell. Instead, BF-TEM tomography was applied. Images were acquired every 2° while tilting over the largest possible range (e.g. -76° to + 76°) which is limited by the microscope setup. After alignment the 2D images were reconstructed by the SIRT (Simultaneous Iterative Reconstruction Technique) algorithm in Astra Toolbox.[4] Due to the missing wedge, artefacts occur in the reconstruction. Because we were able to tilt over a large range, the artefacts are limited. In addition, diffraction contrast is present in the

BF-TEM images. Note that the contrast in the slices through the reconstruction (orthoslices) is inverted in comparison to the BF-TEM tomography series. This contrast inversion is required by the Astra Toolbox to reconstruct the series.

Cell uptake

HeLa cells were plated in a 16-well glass slide at 1 x 10^5 cells/mL, 100 μL cells/well. After overnight incubation, media was replaced with media containing 23 nm nanoparticles at a final concentration of 2 x 10^{11} and 2 x 10^{10} nanoparticles/mL. Cells were incubated with particles for 6 hours, followed by washing with phosphate buffer saline (PBS) and fixation (2% formaldehyde in PBS). Cells were mounted with a coverslip and mounting media (Dako) and photographed the following day.

Characterization of nanoparticles

UV-Vis spectra were recorded using an Agilent 8453 UV-Vis diode-array spectrophotometer. The absorbance spectra were normalized to facilitate comparison. For the thermosensitive samples, an HP 89090A temperature controller was used to heat and cool the samples in the range of 25.0 to 40.0 °C. Measurements were performed 3 minutes after reaching constant temperature. Each heating/cooling cycle was repeated 3 times.
Dynamic light scattering (DLS) experiments were performed in a Zetasizer Nano ZS from Malvern Instruments Ltd., which is equipped with a 4 mW He-Ne laser (wavelength = 632.8 nm), at a set angle of 173 °. The intensity autocorrelation functions were analyzed using the instrument software and the constrained regularization REPES (regularized positive exponential sum) algorithm.[5] Samples were left to equilibrate in the sample holder at a constant temperature for 5 minutes before analyses, which were performed in triplicate.
SAXS curves were obtained at SAXS1 beamline of Brazilian Synchrotron Laboratory (LNLS) in Campinas, Brazil, with a fixed X-ray wavelength of 1.55 Å. Samples were placed in a mica cell window with controlled temperature. Fit2D software was used to integrate CCD images and to subtract parasitic background and solvent. SASfit software version 0.93.5 was used for the fitting procedure.
^{1}H and NOESY NMR spectra were acquired using a Bruker 500 MHz spectrophotometer. Nuclear Overhauser Effect Spectroscopy (NOESY) is a bidimensional NMR technique (2D ^{1}H – ^{1}H) that probes internuclear distances between by means of the nuclear Overhauser effect.[6] The result is a bidimensional spectra, which shows cross peaks between protons that are in close proximity (distance smaller than 0.5 nm) due to cross-correlation. The most intense peak for each polymer was followed, being the ones with chemical shift at 3.6 ppm for PEG, 1.6 ppm for PS and 1.1 ppm for PNIPAM. We used yellow circles to indicate the expected position of cross-peaks if the PEG and PS or PEG and PNIPAM were close. The absence of peaks in those positions indicates that the polymers are probably segregated in the nanoparticle surface.[7,8]
Dark-field (DF) images were obtained with a Nikon Eclipse Ti-U microscope with dark-field oil condenser was used with a x60 S Plan Fluor objective (N.A 0.7) or a x100 oil Plan Fluor objective (N.A 0.5-1.3). Neutral density (ND) and neutral colour balance (NCB) filters were inserted. Photos were taken using a 0.1 s exposure and light intensity levels and other variables were unchanged for the different samples.

Preparation of chloroform/water emulsions

The chloroform/water emulsions were prepared using a mixture of the solvents chloroform:water in 1:9 volume ratio. In a typical experiment, 50 microliters of chloroform was added to 450 microliters of an aqueous solution of PEG 1 kDa + PS 2 kDa Janus nanoparticles ([Au^0] = 1 mM). Straightaway after adding the chloroform a purple and homogenous emulsion was formed, with almost no shaking of the emulsion. The emulsion was studied by UV-vis spectroscopy and DLS with no change in the concentration. The emulsion was diluted five times before placing a 5 microliters drop on TEM grids for TEM experiments.

2. Assembly of Janus Au Nanoparticles with different core sizes

Gold nanoparticles with different values of core size and polymer length exhibit different assembly behavior. The length of the PEG polymer required for stabilizing the Au NPs increases with the increasing diameter of the Au core. Au nanoparticles with a diameter of 33 nm fully coated by PEG 1 kDa assemble (Figure S1C). A coating of the same Au NPs by PEG 1 kDa + PS 2 kDa leads to irreversible aggregation. In the case of Au NPs with a diameter of 13 and 20 nm, the coating with PEG 5 kDa + PS 2 kDa leads to the formation of smaller clusters than for the nanoparticles coated by PEG 1 kDa + PS 2 kDa.

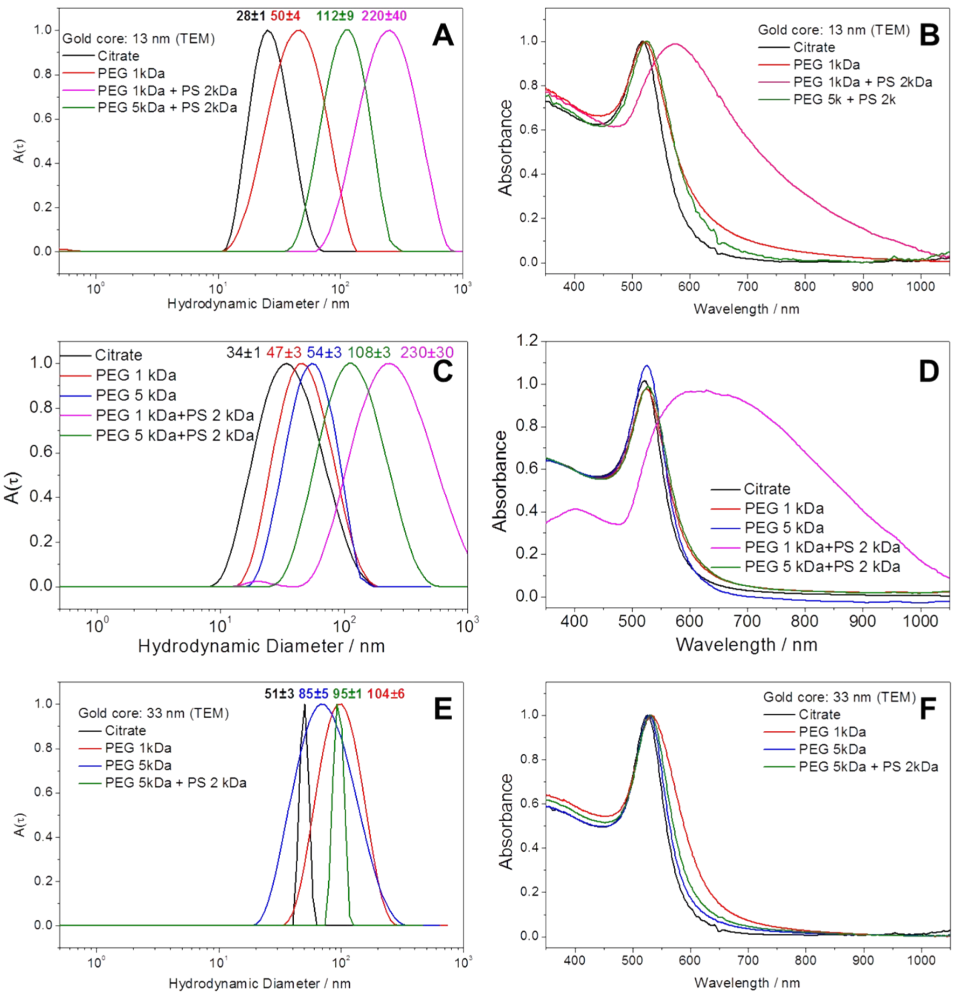

Figure S1. Hydrophilic and PEG+PS Janus nanoparticles (size values obtained from transmission electron microscopy) dispersed in water. The polymer coating is indicated in the figure. A) Size distributions obtained by DLS of samples with a core of 13 nm. B) UV-Vis spectra of samples with a core of 13 nm. C) Size distributions obtained by DLS of samples with a core of 23 nm. D) UV-Vis spectra of samples with a core of 23 nm. E) Size distributions obtained by DLS of samples with a core of 33 nm. F) UV-Vis spectra of samples with a core of 33 nm.

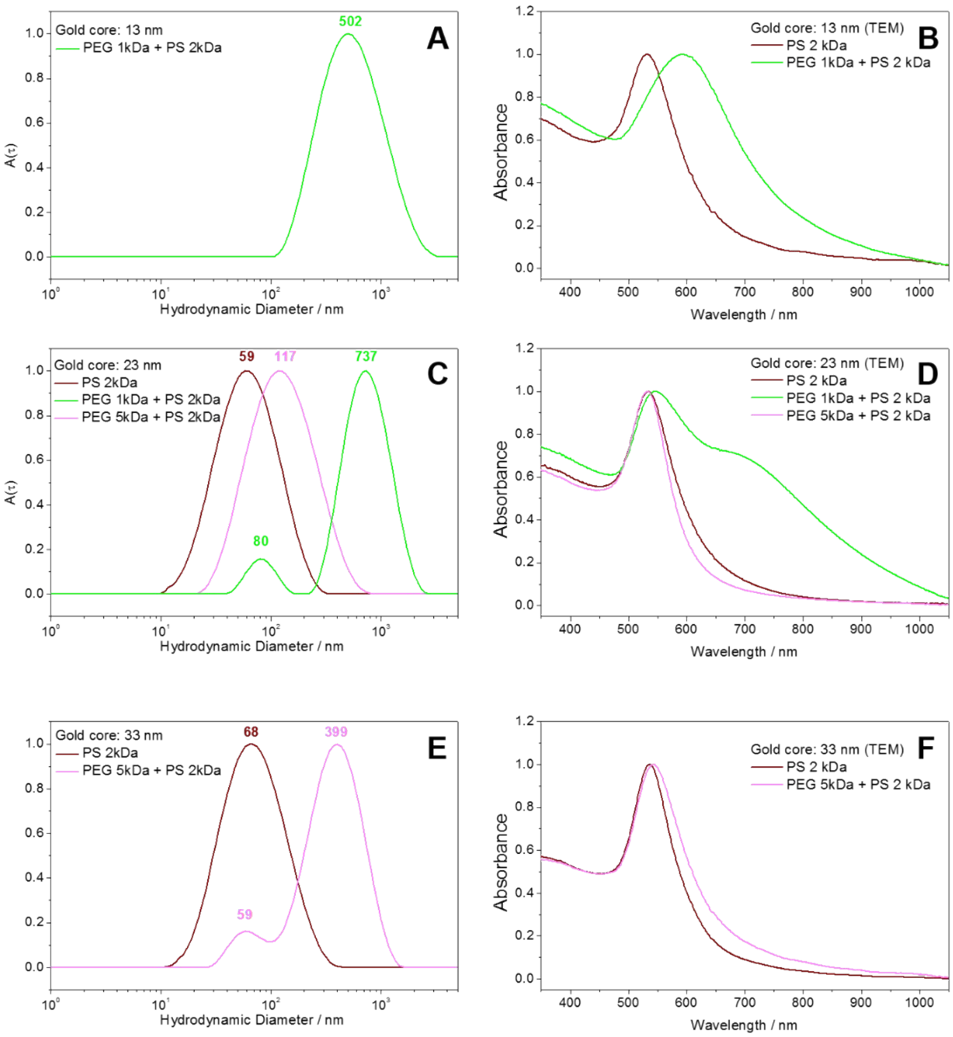

Figure S2. Hydrophobic and Janus nanoparticles (obtained from transmission electron microscopy) dispersed in THF. The polymer coating is indicated in the figure. A) Size distributions obtained by DLS of samples with a core of 13 nm. B) UV-Vis spectra of samples with a core of 13 nm. C) Size distributions obtained by DLS of samples with a core of 23 nm. D) UV-Vis spectra of samples with a core of 23 nm. E) Size distributions obtained by DLS of samples with a core of 33 nm. F) UV-Vis spectra of samples with a core of 33 nm.

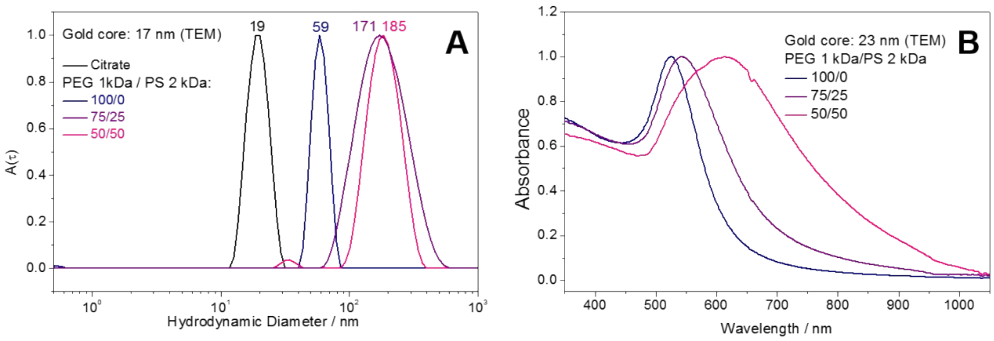

Figure S3. Size and plasmonic features of nanoparticles dispersed in water with coatings of different ratios of added chains of PEG 1 kDa/PS 5 kDa. A) Size distributions obtained by DLS of samples with a core of 17 nm. B) UV-Vis spectra of samples with a core of 23 nm.

20 nm Au NPs coated by PEG 1 kDa + PS 2 kDa were analyzed by SAXS (Figure S4). A curve of the nanoparticles stabilized in citrate and used as gold core for polymer coating is presented for comparison. The experimental curve of the citrate-capped gold nanoparticles was fitted to a model of form factor, $P(q)_{SP}$, of spheres with radius of 11 ± 1 nm. The SAXS curve of nanoparticles coated by PEG 1 kDa and PS 2 kDa exhibit the same form factor, but also the structure factor, $S(q)_{HS}$, observed as an interference peak at q = 0.27 nm^{-1}, which corresponds to a correlation distance of d = 23 nm. Given the nanoparticles radius is 11 nm and the distance between the centers of neighbor scatterers are 23 nm, the nanoparticles are in close contact (gaps of around 1 nm) and the structure factor can be described by a model of hard spheres. However, this model does not explain the high intensity that decays at low q-values. This decay is probably due to the scattering contribution from large objects that are the clusters formed by assembled nanoparticles, as verified by DLS. Therefore, the SAXS curve of NPs coated by PEG 1 kDa and PS 2 kDa is best fitted by the same form factor of nanospheres used for citrate-capped gold nanoparticles, $P(q)_{SP}$, and the structure factor for hard spheres with a volume fraction of 0.345, $S(q)_{HS}$, multiplied by the form factor of large spheres corresponding to the magnitude of the formed clusters, $P(q)_{CL}$:

$$I(q) = n \cdot P(q)_{SP} \cdot S(q)_{HS} \cdot P(q)_{CL}$$

where *n* is the number density of scattering particles. The experimental and fitted curves and the respective parameters obtained are presented in Figure S4 and Table S1.

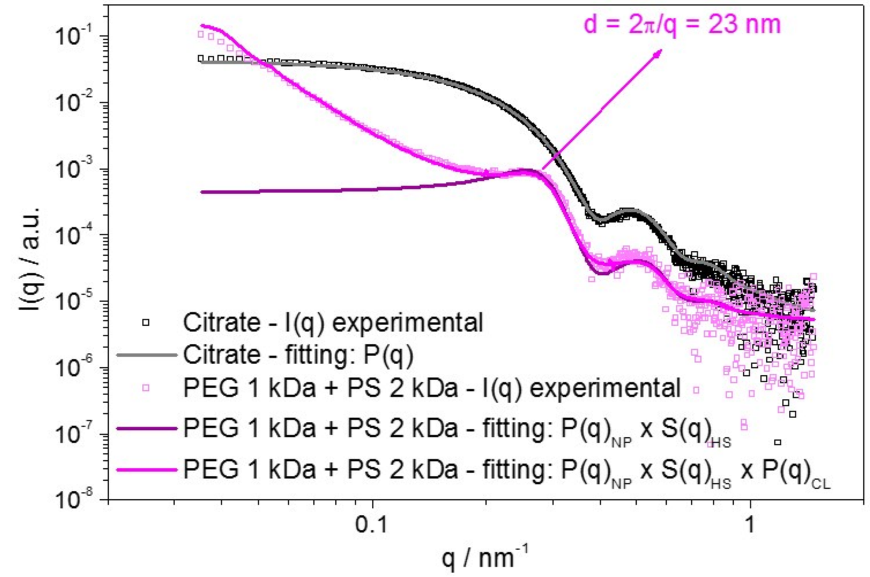

Figure S4. SAXS experimental curves (squares) and fittings (continuous lines). 23 nm Au NPs coated by ligands indicated in the figure.

Table S1: Fitting parameters of SAXS curves of Au nanoparticles (diameter of ca. 20 nm) before and after coating with PEG 1 kDa + PS 2 kDa.

Factor	Parameter	Citrate stabilized nanoparticles	Janus nanoparticles (PEG+PS)
P(q)SP	Radius of nanoparticles (nm)	11.1	11.1
	Standard deviation in a Gaussian distribution (nm)	1.3	1.3
$S(q)_{HS}$	Hard sphere repulsion radius (nm)	-	11.1
	Volume fraction	-	0.345
$P(q)_{CL}$	Radius of clusters of nanoparticles (nm)	-	218
	Standard deviation in a Gaussian distribution (nm)	-	26

3. Chloroform/Water Emulsions stabilized by Janus Au NPs

Janus nanoparticles are expected to increase interfacial activity, thus being promising toward formation of Pickering emulsions. Sonication of Janus AuNPs dispersed in chloroform, in the presence of water, leads to emulsification. The obtained emulsions were analyzed by UV-Vis spectroscopy, dynamic light scattering and transmission electron microscopy to evaluate the sizes of the formed drops and their stability (Figures S5, S6).

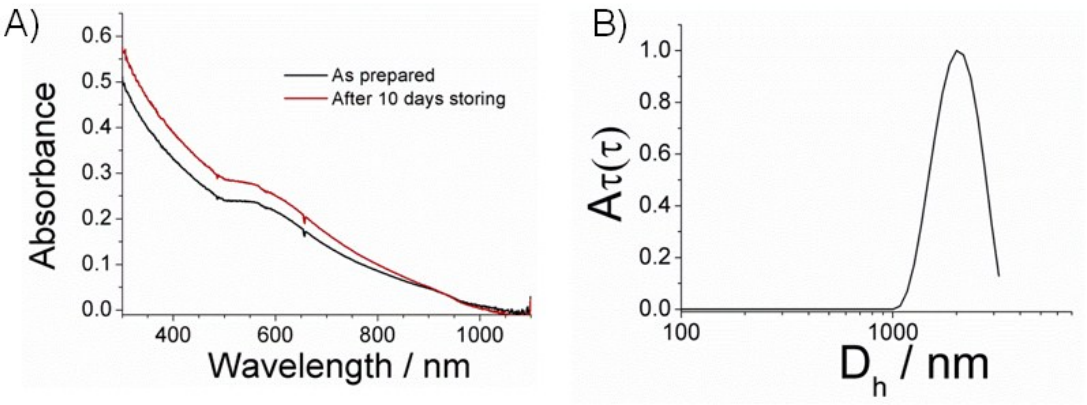

Figure S5. Chloroform/water emulsions with Janus Au nanoparticles. **A)** UV-Vis spectra for the emulsion as prepared (black line) and after storage at RT under light ambient conditions for 10 days (red line) **B)** Hydrodynamic diameter measured by DLS.

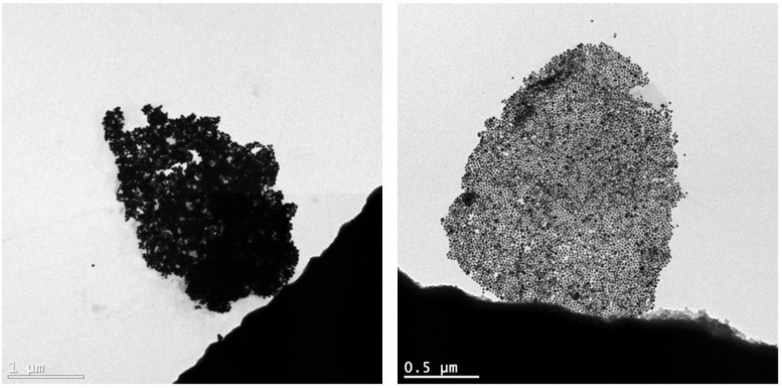

Figure S6. TEM images of dried emulsions. Drop casted chloroform/water emulsions with Janus Au nanoparticles on TEM grid.

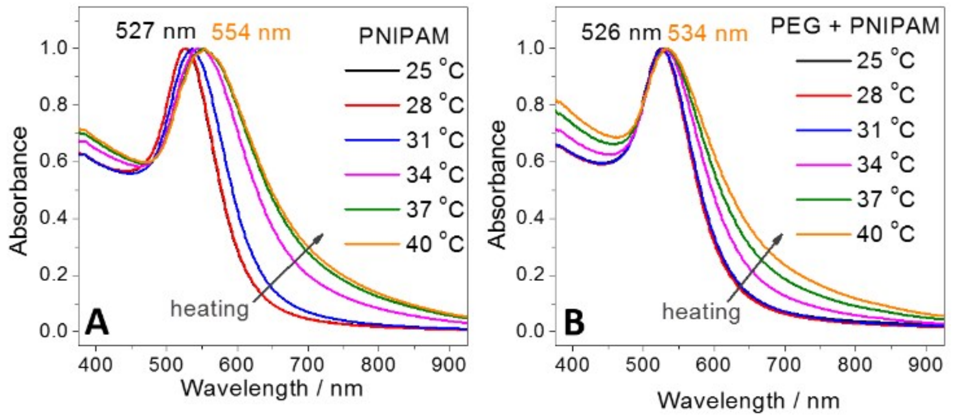

Figure S7. UV-Vis spectra at different temperatures for 20 nm Au NPs coated by: A) PNIPAM 1.2kDa; B) PEG 1kDa + PNIPAM 1.2kDa.

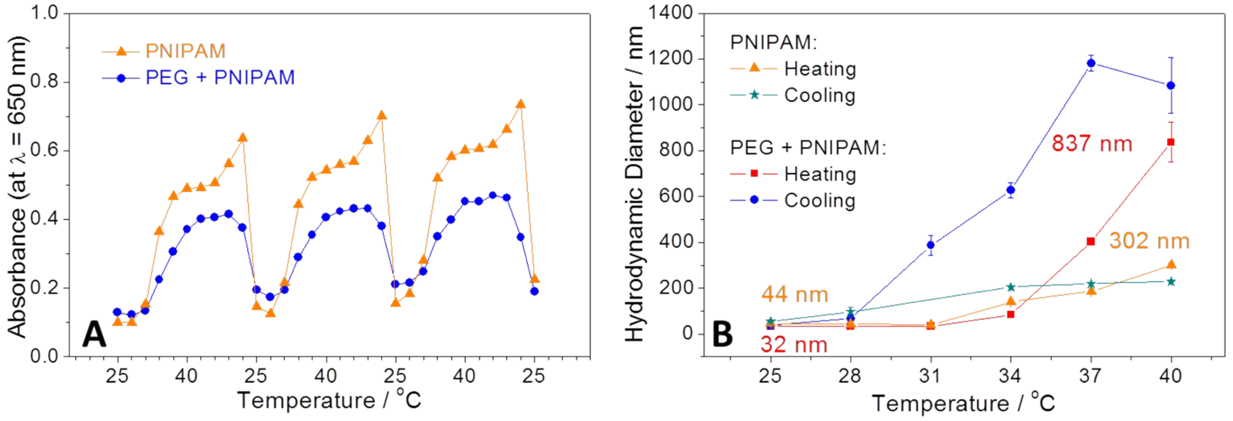

Figure S8. Characterization at different temperatures for 20 nm Au NPs coated by PNIPAM 1.2kDa and PEG 1kDa + PNIPAM 1.2kDa. **A** – Absorbance values at 650 nm; **B** – Hydrodynamic diameter.

4. 3D investigation of Janus Au NPs coated by PEG+PS

A study of the shell stability using the nanoparticles coated only by one of the two polymers shows that the stained shell is initially observed for both cases. However, for nanoparticles coated only by PNIPAM, the shell disappears after a few seconds to minutes of irradiation with the electron beam.

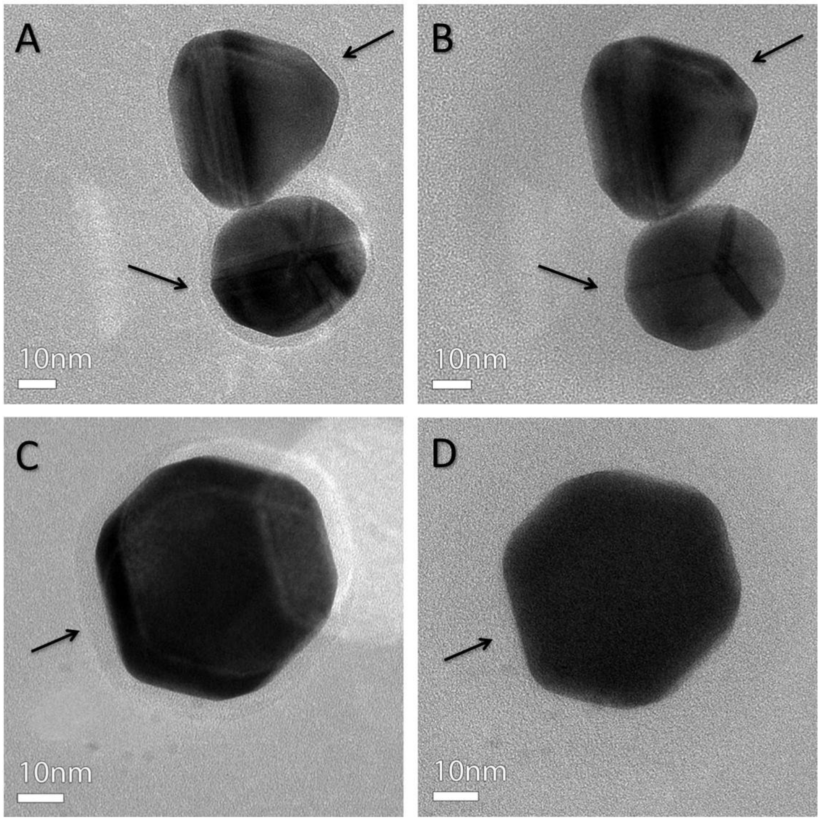

Figure S9. BF-TEM images of AuNPs coated by PNIPAM 1.2 kDa and stained with $CuSO_4$. After a few seconds to minutes and a few acquisitions, the stained shell in Figures A and C disappears as shown in B and D.

The shell is stable enough to be analyzed by electron tomography in the case of PEG-coated Au NPs. For Au NPs coated by PEG+PNIPAM, a half shell is observed, which is expected to be the coating corresponding to PEG.

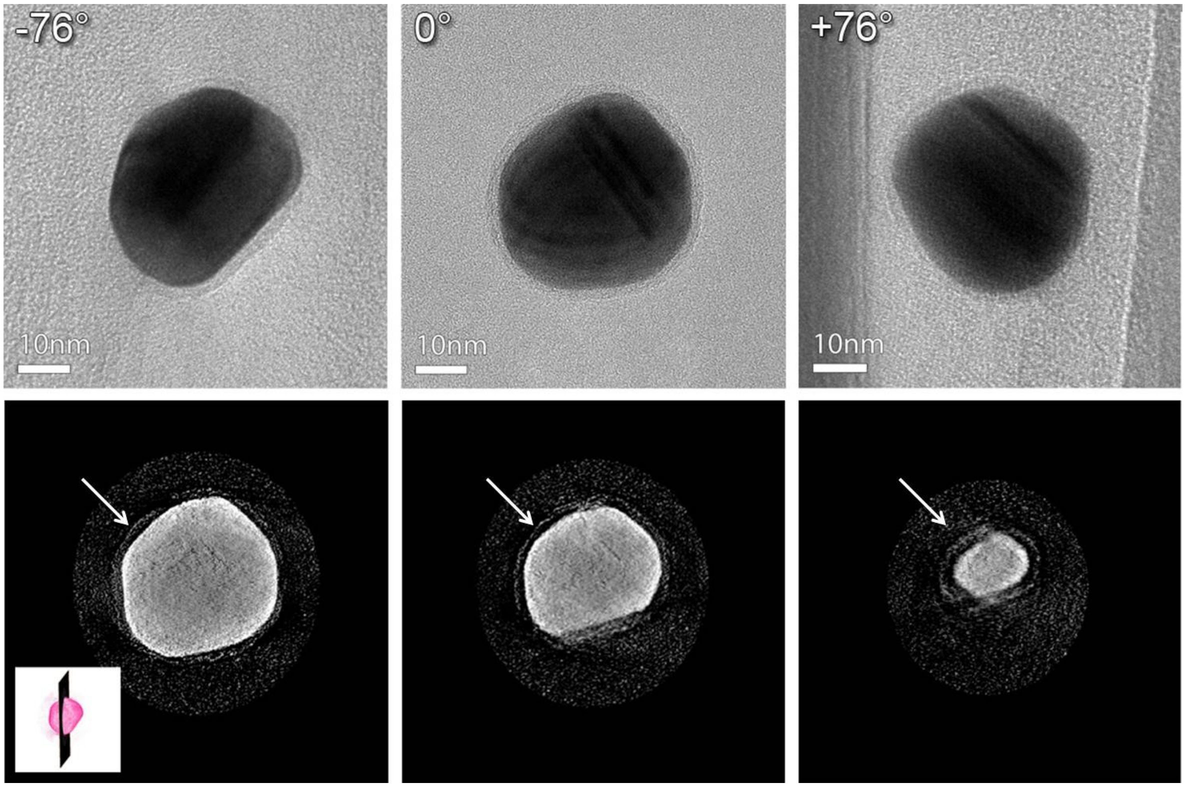

Figure S10. BF-TEM images of AuNPs coated by PEG 1 kDa and stained with $CuSO_4$. A complete shell is observed in every 2D projection (first row). The tilt series was reconstructed with the SIRT algorithm (Astra toolbox [4]). In the orthoslices (second row), a complete shell is observed around the particle.

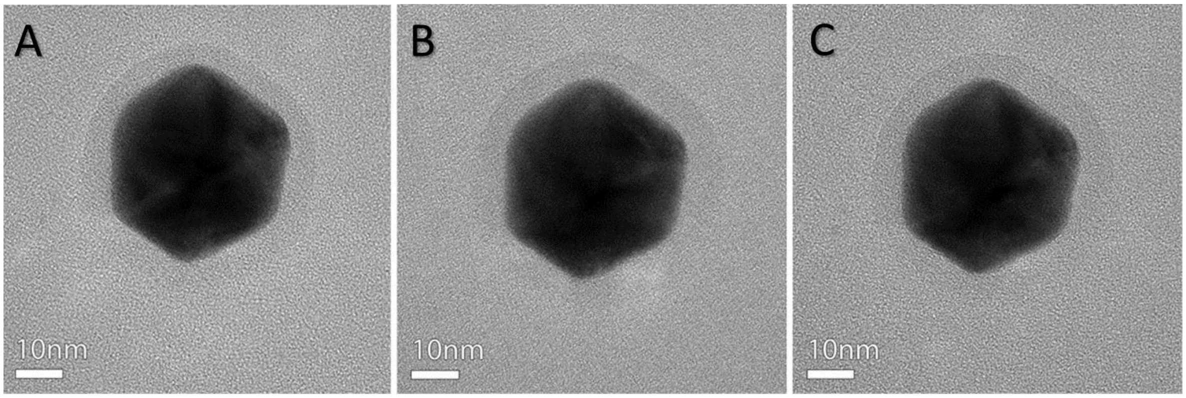

Figure S11. BF-TEM images of AuNPs coated by PEG 1 kDa + PNIPAM 1.2 kDa and stained with $CuSO_4$. Images were acquired at 0 degrees before (A), during (B) and after (C) the acquisition of the tilt series (Figure 2 in the main text). A half shell is observed in the 2D projection. Based on the images, it is clear that the half shell-like structure is maintained and stable.

The following results (Figures S12-S15) were obtained in a similar way. The tilt series was acquired under the same conditions and reconstructed in the same manner. Again a half shell was observed. The results were added to show the existence of a half stained shell in different tilt series.

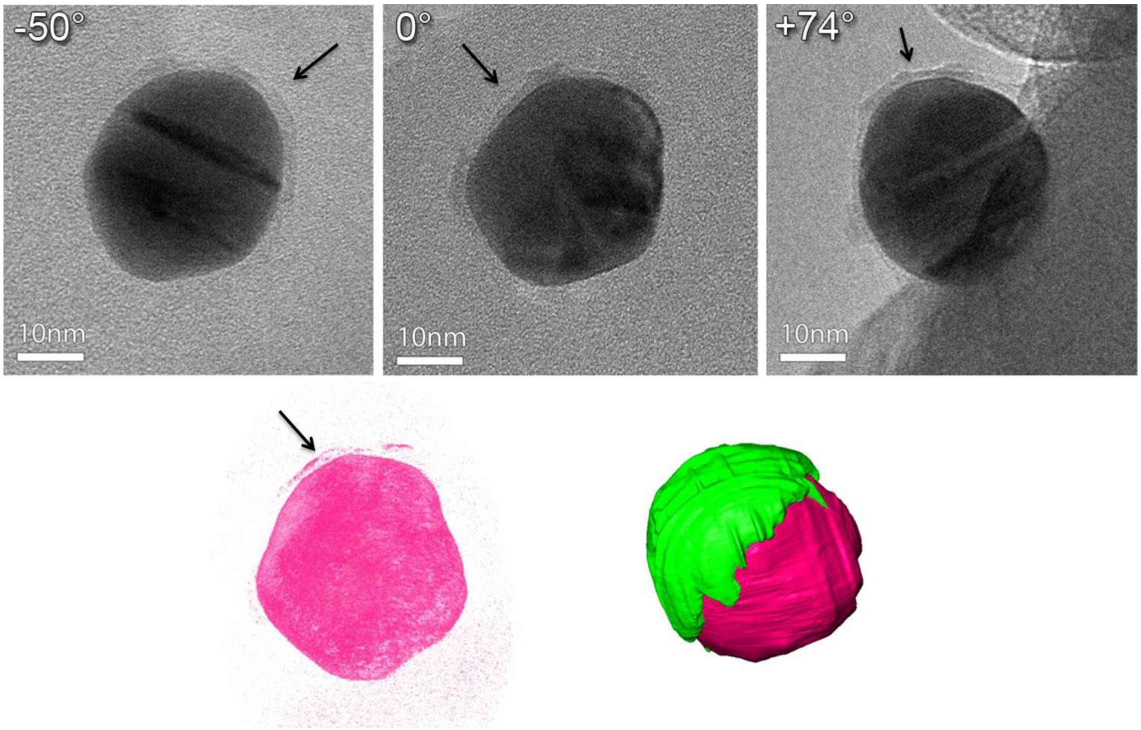

Figure S12. BF-TEM tomography for nanoparticles coated by PEG 1 kDa + PNIPAM 1.2 kDa and stained with $CuSO_4$. At some places the shell is visible in the 3D reconstruction. A manual segmentation could be performed based on the orthoslices.

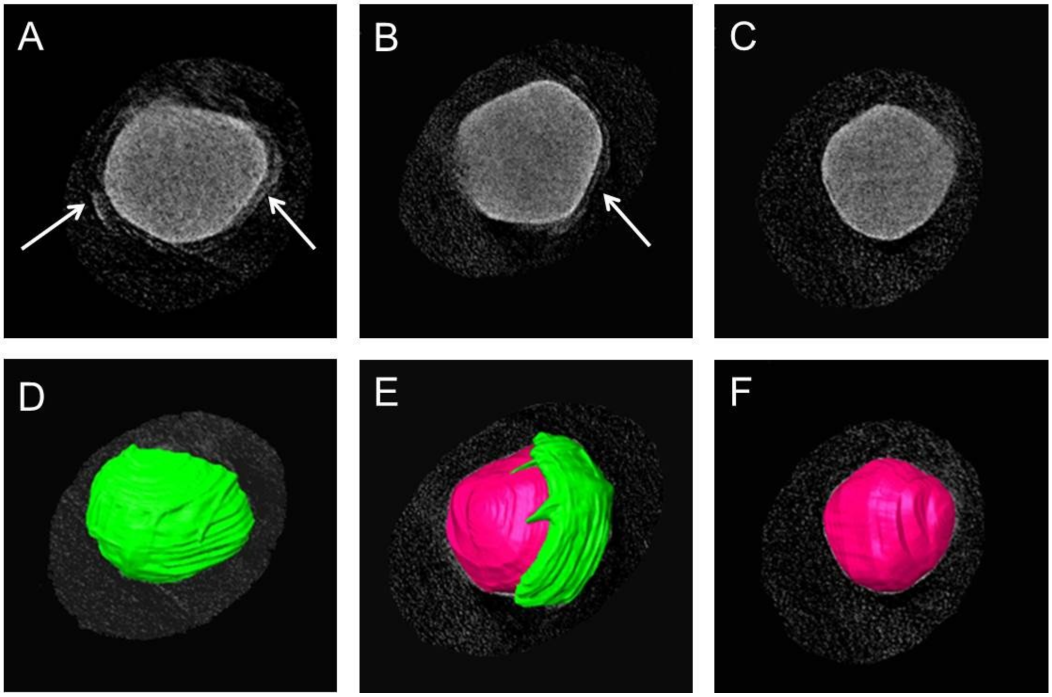

Figure S13. BF-TEM tomography for nanoparticles coated by PEG 1 kDa + PNIPAM 1.2 kDa and stained with $CuSO_4$. Slices through the reconstruction show the absence of a shell (C, F), the presence of a complete shell (A, D) and a half shell (B, E) around the particle (indicated by white arrows) dependent on the position of the slice.

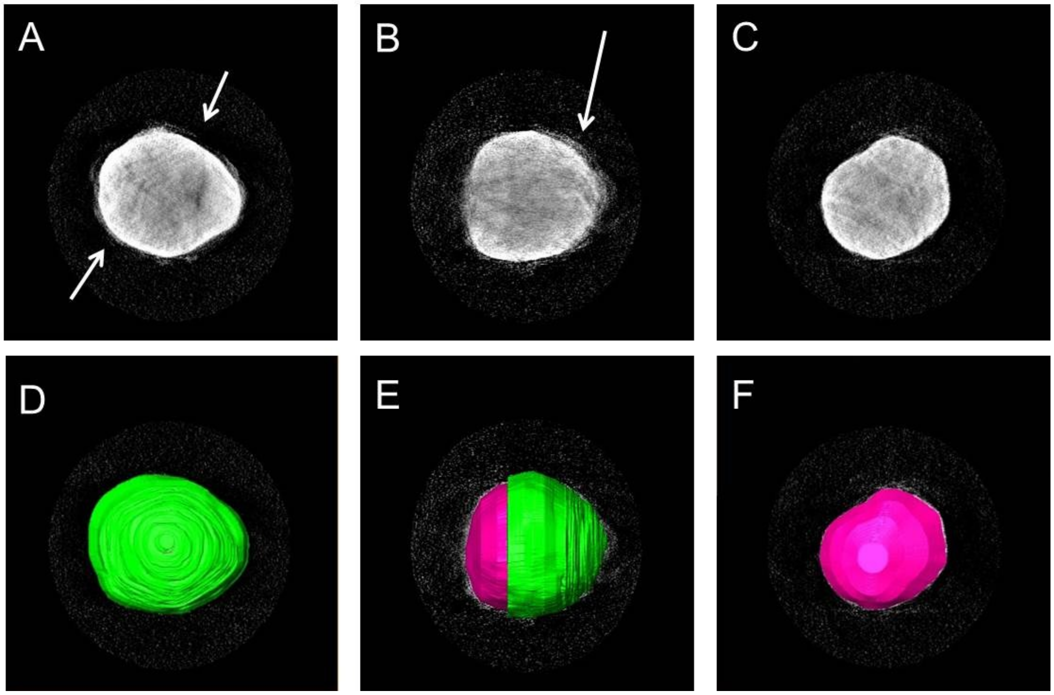

Figure S14. BF-TEM tomography for nanoparticles coated by PEG 1 kDa + PNIPAM 1.2 kDa and stained with $CuSO_4$. Slices through the reconstruction show the absence of a shell (C, F), the presence of a complete shell (A, D) and a half shell (B, E) around the particle (indicated by white arrows) dependent on the position of the slice.

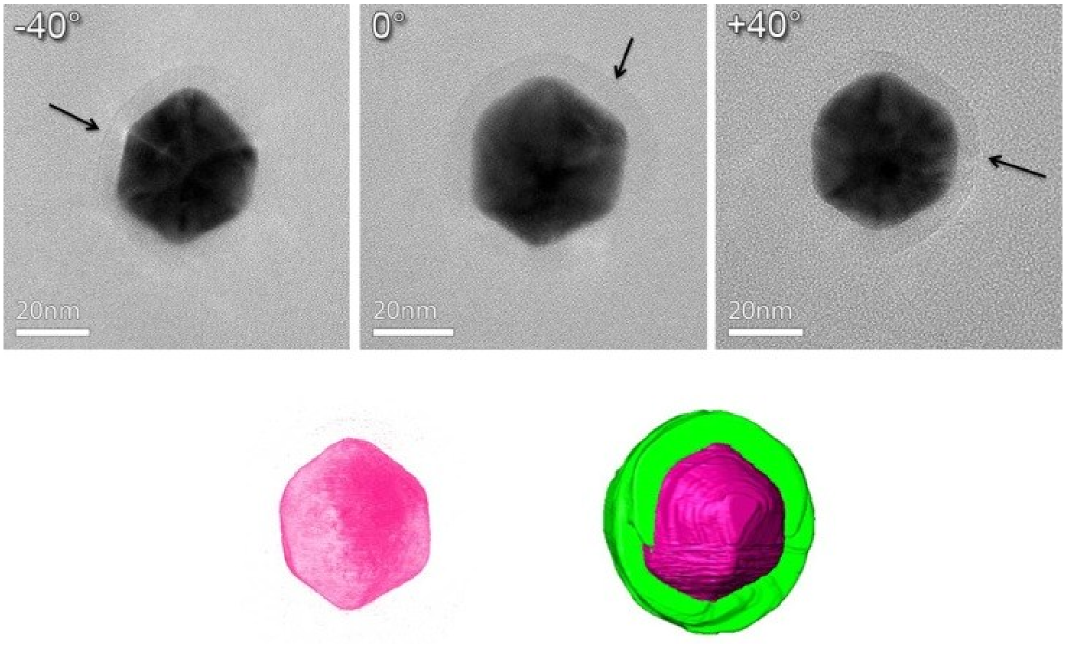

Figure S15. BF-TEM tomography for Au NPs coated by PEG 1kDa + PNIPAM 1.2kDa, stained with CuSO4. Images were acquired from -60º to 76º, every 2 degrees. A (roughly) half shell was observed in every 2D projection of the tilt series. 3D visualization of the reconstructed object and the result of manual segmentation are shown in the bottom row.

Janus Au NPs coated by PEG+PS are investigated by selectively staining one of the polymers. Calcium ions preferably bind to PEG. Therefore, calcium phosphate was used to stain PEG in Janus Au NPs coated by PEG+PS, providing contrast between the two coating polymers. TEM images suggested that one hemi-sphere of the particle is coated by PEG and stained with calcium phosphate. Yet, these pictures only correspond to 2D projections of the Janus Au NPs (Figure S16). However, it is important to obtain 3D structural information on the actual Janus character and therefore electron tomography is performed. After acquisition and reconstruction of a tilt series of images (see Materials and Methods), the results presented in Figure S17 are obtained. The 3D reconstructed object shows low contrast between the shell and the background. Therefore, a manual segmentation based on the orthoslices was performed. Segmentation in the 3 different directions (xy, yz, zx) confirms the formation of a stained half-shell as indicated in green in Figure S17.

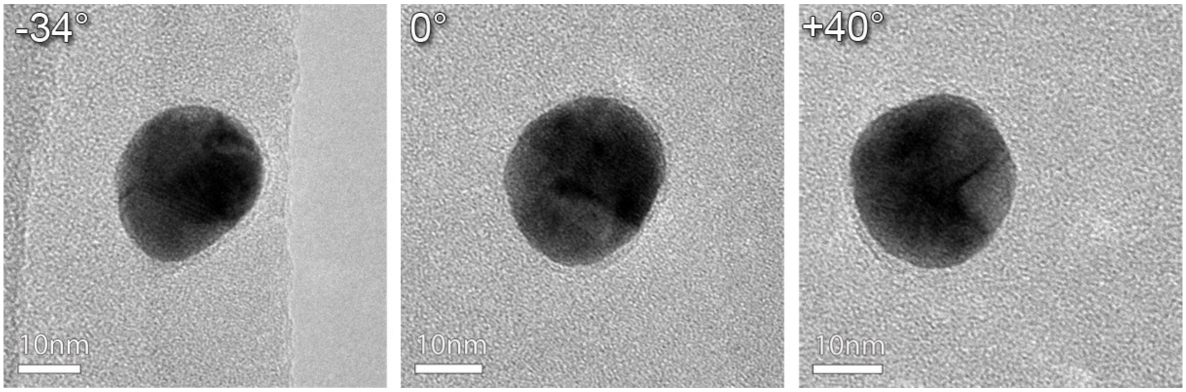

Figure S16. BF-TEM images of Au NPs coated by PEG+PS and stained with calcium phosphate.

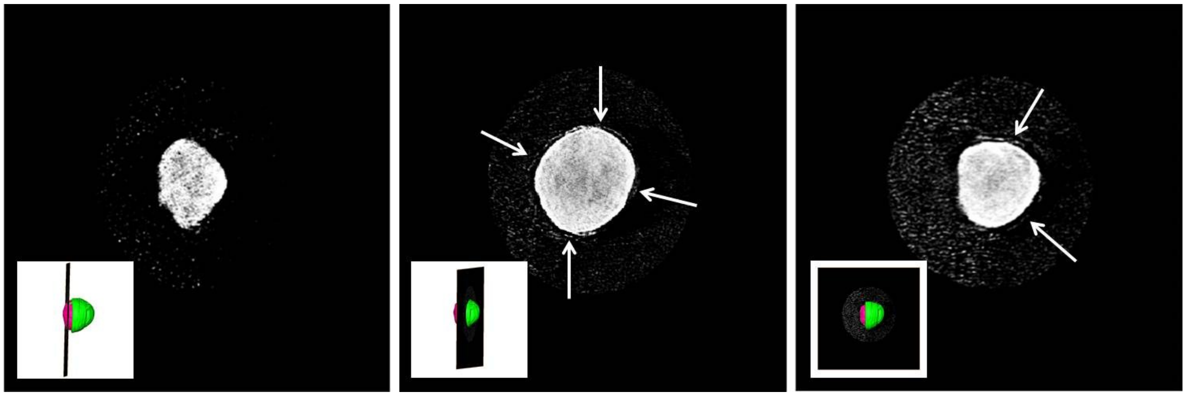

Figure S17. The tilt series was reconstructed using the SIRT algorithm (Astra toolbox [4]). Slices through the reconstruction show the absence of a shell, the presence of a complete shell and a half shell around the particle (indicated by white arrows) dependent on the position of the slice.

5. TEM images of Janus AuNPs after silica growth over PEG half shell

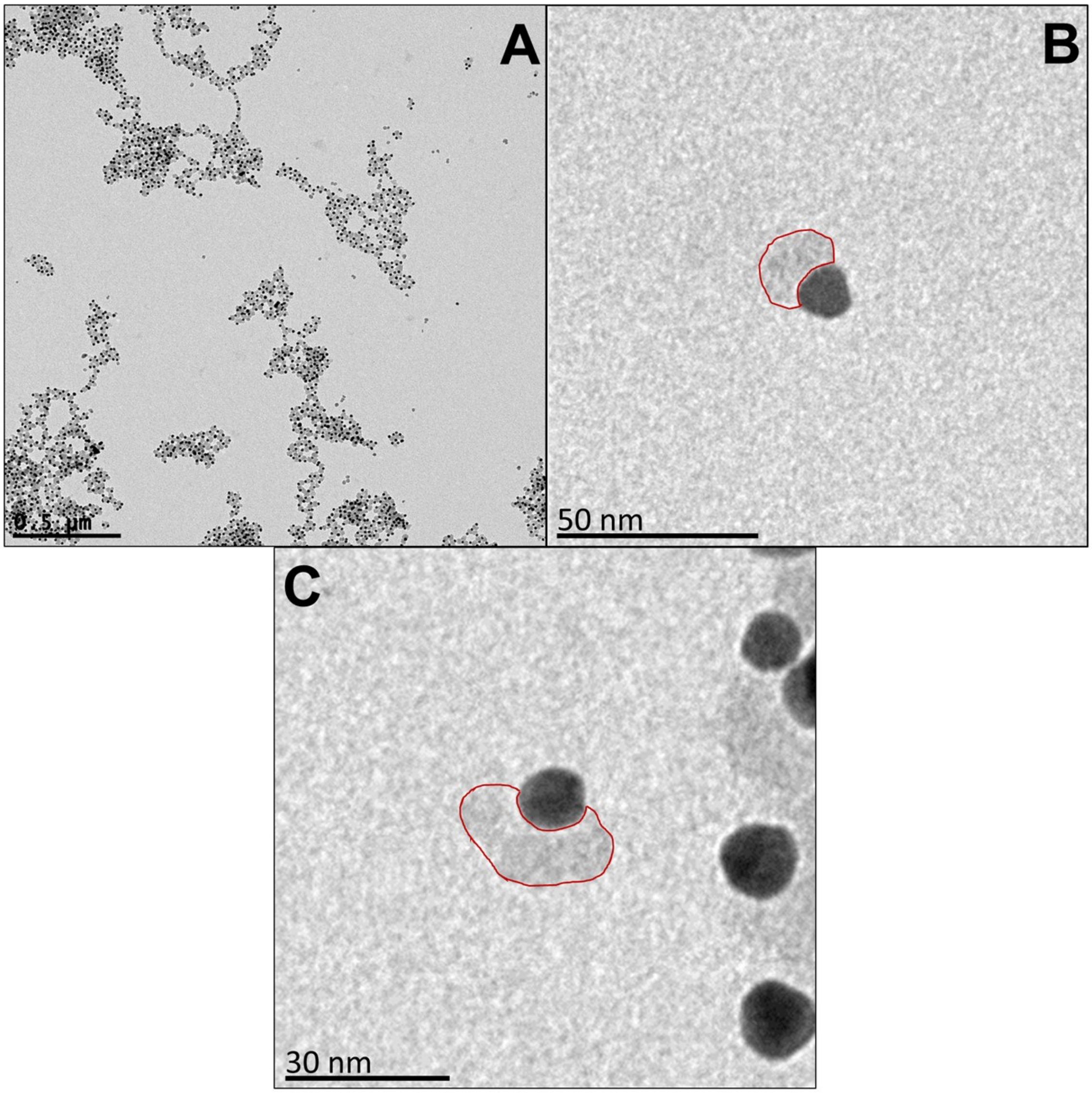

Figure S18. TEM image of 13 nm Au NPs previously coated by PEG 5 kDa + PS 2 kDa after silica growth. Red lines highlight silica half-shells.

References

(1) Bastús, N. G.; Comenge, J.; Puntes, V. *Langmuir* **2011**, *27* (17), 11098.

(2) Enüstün, B. V.; Turkevich, J. *J. Am. Chem. Soc.* **1963**, *85* (21), 3317.

(3) Fernández-López, C.; Mateo-Mateo, C.; Álvarez-Puebla, R. A.; Pérez-Juste, J.; Pastoriza-Santos, I.; Liz-Marzán, L. M. *Langmuir* **2009**, *25* (24), 13894.

(4) van Aarle, W.; Palenstijn, W. J.; De Beenhouwer, J.; Altantzis, T.; Bals, S.; Batenburg, K. J.; Sijbers, J. *Ultramicroscopy* **2015**, *157*, 35.

(5) Jakeš, J. *Collect. Czech. Chem. Commun.* **1995**, *60* (11), 1781.

(6) Voets, I. K.; Keizer, A. De; Stuart, M. A. C.; Waard, P. De. *Macromolecules* **2006**, *39*, 5952.

(7) Liu, X.; Yu, M.; Kim, H.; Mameli, M.; Stellacci, F. *Nat. Commun.* **2012**, *3* (1182), 1.

(8) Kim, H.; Carney, R. P.; Reguera, J.; Ong, Q. K.; Liu, X.; Stellacci, F. *Adv. Mater.* **2012**, *24* (28), 3857.
